# Kv7 potassium channel activation with ICA-105665 reduces photoparoxysmal EEG responses in patients with epilepsy

**DOI:** 10.1111/epi.12224

**Published:** 2013-05-20

**Authors:** Dorotheé G A Kasteleijn-Nolst Trenité, Victor Biton, Jacqueline A French, Bassel Abou-Khalil, William E Rosenfeld, Bree Diventura, Elizabeth L Moore, Seth V Hetherington, Greg C Rigdon

**Affiliations:** * Department of Medical Genetics, Utrecht UniversityUtrecht, The Netherlands; † Department of Neuroscience, Sapienza UniversityRome, Italy; ‡ Arkansas Epilepsy ProgramLittle Rock, Arkansas, U.S.A; § Department of Neurology, New York UniversityNew York City, New York, U.S.A; ¶ Department of Neurology, Vanderbilt UniversityNashville, Tennessee, U.S.A; # Comprehensive Epilepsy Care Center for Children and AdultsSt Louis, Missouri, U.S.A; ** I Epilepsy ConsortiumNew York, New York, U.S.A; †† Icagen, Inc.Durham, North Carolina, U.S.A

**Keywords:** ICA-105665, Kv7 potassium channel, Ion channel, Epilepsy, Photosensitivity, Photoparoxysmal response, Proof of concept model

## Abstract

**Purpose** To assess the effects of ICA-105665, an agonist of neuronal Kv7 potassium channels, on epileptiform EEG discharges, evoked by intermittent photic stimulation (IPS), the so-called photoparoxysmal responses (PPRs) in patients with epilepsy.

**Methods** Male and female patients aged 18–60 years with reproducible PPRs were eligible for enrollment. The study was conducted as a single-blind, single-dose, multiple-cohort study. Four patients were enrolled in each of the first three cohorts. Six patients were enrolled in the fourth cohort and one patient was enrolled in the fifth cohort. PPR responses to 14 IPS frequencies (steps) were used to determine the standard photosensitivity range (SPR) following placebo on day 1 and ICA-105665 on day 2. The SPR was quantified for three eye conditions (eyes closing, eyes closed, and eyes open), and the most sensitive condition was used for assessment of efficacy. A partial response was defined as a reduction in the SPR of at least three units at three separate time points following ICA-105665 compared to the same time points following placebo with no time points with more than three units of increase. Complete suppression was defined by no PPRs in any eye condition at one or more time points.

**Key Findings** Six individual patients participated in the first three cohorts (100, 200, and 400 mg). Six patients participated in the fourth cohort (500 mg), and one patient participated in the fifth cohort (600 mg). Decreases in SPR occurred in one patient at 100 mg, two patients receiving 400 mg ICA-105665 (complete abolishment of SPR occurred in one patient at 400 mg), and in four of six patients receiving 500 mg. The most common adverse events (AEs) were those related to the nervous system, and dizziness appeared to be the first emerging AE. The single patient in the 600 mg cohort developed a brief generalized seizure within 1 h of dosing, leading to the discontinuation of additional patients at this dose, per the predefined protocol stopping rules.

**Significance** ICA-105665 reduced the SPR in patients at single doses of 100 (one of four), 400 (two of four), and 500 mg (four of six). This is the first assessment of the effects of activation of Kv7 potassium channels in the photosensitivity proof of concept model. The reduction of SPR in this patient population provides evidence of central nervous system (CNS) penetration by ICA-105665, and preliminary evidence that engagement with neuronal Kv7 potassium channels has antiseizure effects.

ICA-105665, identified by Icagen, Inc. (Durham, NC, U.S.A.), is a novel small molecule that opens Kv7.2/7.3 and Kv7.3/7.5 potassium channels (Roeloffs et al., 12), also known as KCNQ2/3 and KCNQ3/5 channels. The compound has demonstrated broad spectrum antiseizure activity in multiple animal models including maximal electroshock, 6 Hz seizures, pentylenetetrazole, and electrical kindling at doses from <1 to 5 mg/kg (Roeloffs et al., 12). ICA-105665 was well-tolerated in both a single ascending dose study in healthy volunteers at doses up to 400 mg (Risner et al., 11) and in healthy volunteers and patients with epilepsy when administered twice daily in a 7-day repeat dose study at total daily doses up to 600 mg (Hetherington et al., 3; Rigdon et al., 9).

The photosensitivity epilepsy model provides a means of assessing the effects of potential antiepileptic drugs (AEDs) in patients in a controlled laboratory setting. Photosensitivity describes the ability to produce an epileptiform electroencephalography (EEG) response evoked by intermittent photic stimulation (IPS). This EEG pattern is called a photoparoxysmal response (PPR).

Administration of approved and experimental AEDs, as single or repeated doses, clearly diminishes or even abolishes the response to IPS (Binnie et al., 1). A standardized method for eliciting PPR in response to IPS has been devised to quantify the effects of promising new therapies for epilepsy (Kasteleijn-Nolst Trenité et al., 7). This approach has successfully identified a number of currently approved AEDs as well as several molecules under investigation including lamotrigine (LTG), levetiracetam (LEV), vigabatrin (VGB), brivaracetam (BRV), and carisbamate (Binnie et al., 2; Rimmer et al., 10; Kasteleijn-Nolst Trenité et al., 5, 6; Trenité et al., 14). So far only one experimental drug, an *N*-methyl-d-aspartate (NMDA) full antagonist, Org6370, has proven not to be suppressive, but proconvulsive in a dose-dependent way (Kasteleijn-Nolst Trenité et al., 4). This assay allows investigators to establish that the compound under study is penetrating to the central nervous system (CNS) compartment and engaging the target channel or receptor under study by suppressing the epileptiform EEG discharges.

The objectives of the current study were the following: (1) to assess the effects of ICA-105665 on the IPS-induced photoparoxysmal EEG response in patients with epilepsy; (2) to correlate plasma concentrations of ICA-105665 with pharmacodynamic effects; and (3) to assess the safety of a single dose of ICA-105665 in patients with photosensitive epilepsy.

This was the first evaluation of the effects of activation of Kv7 potassium channels on the photic-induced epileptiform reactions and the first proof of concept (POC) epilepsy trial in humans of ICA-105665.

## Methods

### Patients

Patients between 16 and 60 years of age with a minimum weight of 46 kg and a diagnosis of epilepsy for which they were taking 0–2 concurrent AEDs and otherwise healthy were enrolled in the study. Patients had to exhibit a reproducible PPR on EEG of at least three points on a frequency assessment scale with no change of more than three frequencies in two repeated measurements recorded at the Screening Visit.

### Study design, randomization, and blinding

This was a phase 2, multicenter, single-blind (investigator open, patient blinded), single-dose, placebo-controlled multiple-cohort study of the pharmacodynamic effect of ICA-105665 at different dosages on the EEG response to IPS in patients with epilepsy. Each patient received both placebo and active treatment on consecutive days. Because of the difficulty in finding these patients, due to age restrictions and hospital stay for three consecutive days, no sample size calculations were conducted and the cohort size of 4–6 patients was based on practical and historical considerations. The institutional review board of each investigator's institution approved the study protocol. All procedures were performed in accordance with the International Conference on Harmonization of Technical Requirements for Registration of Pharmaceuticals for Human Use—Good Clinical Practice (ICH-GCP) guidelines. Finally, each subject gave his/her written informed consent.

**Figure 1:**
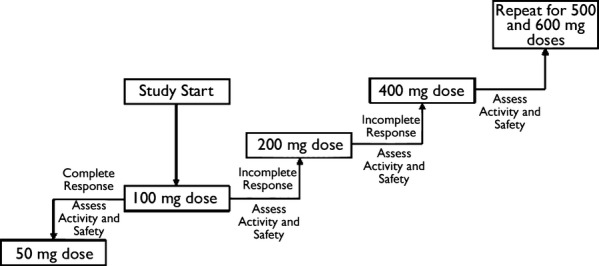
Study schematic. Epilepsia © ILAE

A schematic diagram of the study conduct is presented in Fig. 1. After each cohort was completed, safety and activity was assessed by a committee including the study investigators, the project medical monitor, and the blinded EEG reader prior to allowing enrollment of the next cohort of patients.

### Efficacy

After obtaining informed consent, patients were screened within 21 days prior to enrollment. Each dose cohort consisted of four patients (cohorts receiving 100, 200, and 400 mg ICA-105665) or six patients (cohorts receiving 500 and 600 mg ICA-105665).

On day 1, patients received placebo and IPS assessments to establish the baseline frequency range of their response. IPS was delivered during three eye conditions: eyes closing, eyes closed, and eyes open. The procedure was stopped as soon as generalized epileptiform discharges were evoked. The following flash frequencies were tested: 2, 5, 8, 10, 13, 15, 18, 20, 23, 25, 30, 40, 50, and 60 Hz. The lowest and highest frequencies eliciting a PPR defined the range of the standard photosensitive response (SPR). For example, if the first flash frequency eliciting a PPR when going from low to high frequency was 8 Hz and the first frequencies eliciting a PPR when going from high to low frequencies was 25 Hz, then the SPR was 8 units. The maximum possible SPR was 14 units.

On day 2, patients received a single oral dose of ICA-105665 after the first baseline IPS and IPS assessments were again performed as on day 1. A patient was considered to have had a positive response if either of the following occurred:

1. Complete suppression of PPR (photosensitivity) as demonstrated by suppression in all eye conditions at one or more time points, OR.
2. A reduction of the SPR of at least three units (equivalent to a change compared to the baseline [day 1] IPS assessment of three frequency steps between the upper and lower frequency response limits) at three separate time points, compared with the same time points on day 1 or predose day 2, and no time points with three units of increase.

SPR was assessed at predose, 1, 2, 3.5, 5, 6.5, and 8 h post placebo (day 1) or ICA-105665 (day 2). On day 3 SPR was assessed 24 and 28 h post day 2 dose.

Reading of the EEG studies for the final determination of the SPR was performed by an independent and blinded reader (DKNT) who was not one of the clinical site investigators. Coded data were sent to Dr. Kasteleijn who scored the EEG trace following each IPS and then determined the SPR. The blinding code was broken only after she had completed reading all data from each subject.

Patients were allowed to participate in one or more cohorts, with a minimum time of 4 weeks between day 1 for each cohort of participation.

### Safety and tolerability

Safety was evaluated by assessment of adverse events, laboratory tests, vital signs, physical and neurologic examinations, and 12-lead electrocardiography.

### Pharmacokinetics

Plasma samples were collected from each patient at trough at Screen and day −1 for concomitant AEDs, and then on day 2 predose, and 1, 2, 3.5, 5, 6.5, and 8 h postdose for analysis of concomitant AEDs and ICA-105665 concentrations. On day 3, 24 and 28 h postdose plasma samples were obtained. Samples were analyzed by high-performance liquid chromatography (HPLC) with tandem mass spectrometry (MS/MS) detection by Covance Bioanalytical Services, LLC (Indianapolis, IN, U.S.A.). Noncompartmental pharmacokinetic analysis and some descriptive statistics were derived with WinNonlin Professional version 5.2 (Pharsight Corp, Mountain View, CA, U.S.A.) by Icagen, Inc.

### Statistical analysis

Two analysis populations were defined for this study. In general, disposition, demographics, other baseline characteristics, and all safety data were summarized using the Safety Population, whereas all analyses relative to PPR utilized the modified intent-to-treat (mITT) population.

1. The Safety population was based on all patients who received at least one dose of study medication (ICA-105665 or placebo).
2. The mITT population included all patients who received at least one dose of study medication and had a defined SPR on at least one time point on study day 2.

Appropriate descriptive statistics were computed and displayed for both categorical and continuous variables. Basically, SPR data on day 2 (Drug) were compared with those on day 1 (Placebo). Results on days 2 and 3 allowed us also to determine the duration of suppression of the SPR. Statistical analysis was conducted using Version 9.1.3 or later of the SAS software package by StatWorks, Inc. (Research Triangle Park, NC, U.S.A.).

## Results

### Patient characteristics and disposition

Overall, 13 patients participated in the study, 4 of whom enrolled in more than one dose cohort: 4 patients each in the 100, 200, and 400 mg cohorts; 6 patients in the 500 mg cohort; and 1 patient in the 600 mg cohort (for a total of 19 dose cohort enrollments by 13 individual patients). The patients who enrolled in more than one dose cohort can be summarized as follows: one patient in the 100- and 200-mg dose cohorts; two patients in the 100-, 200-, and 400-mg dose cohorts; one patient in the 200- and 400-mg dose cohorts. One patient (600 mg) was discontinued from the study because of a generalized tonic–clonic seizure on day 2.

The mean (standard deviation, SD) age was 29.8 (11.36) years. Most patients were female (69%), and white (100%). Overall mean (SD) baseline characteristics were the following: height, 166.45 (9.443) cm; weight, 73.31 (16.344) kg; and body mass index [BMI], 26.38 (5.282) kg/m^2^.

All 13 patients had idiopathic generalized epilepsy (IGE): 6 had juvenile myoclonic epilepsy, 1 had childhood absence, and 6 were not otherwise specified. By history of seizure type, there were 11 patients (85%) with generalized tonic–clonic seizures, 9 (69%) with absences and 7 patients (54%) with myoclonic seizures.

Two patients were without concomitant AEDs; the others took either monotherapy with carbamazepine (CBZ) or topiramate (TPM) or polytherapy with levetiracetam (LEV) and lamotrigine (LTG), LEV and clonazepam (CLZ), phenobarbitone (PB) and phenytoin (PHT), with all serum levels within normal limits.

### Efficacy

The most sensitive eye condition was defined as the condition that yielded the largest SPR on day 1 before dosing with placebo. In the event of a tie, the condition that yielded the largest average SPR across all time points on day 1 was the most sensitive condition. The most sensitive eye condition appeared to be Eyes Closing (two subjects, 100 mg; four subjects, 200 mg; three subjects, 400 mg; five subjects, 500 mg).

**Figure 2:**
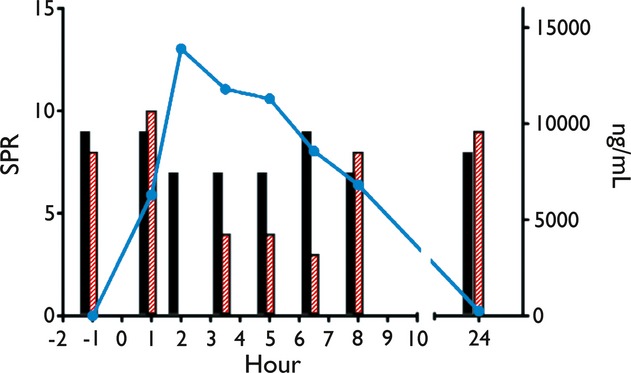
Plot depicting response to IPS and plasma concentrations of ICA-105665 for a single subject dosed at 400 mg. Filled bars represent SPR on day 1 (placebo) and hatched bars represent SPR on days 2 and 3 (ICA-105665). An exception is the black bar plotted at the 24 h time point. For comparative purposes this SPR value is from predose on day 2 and is therefore represented twice on the plot (predose as a hatched bar and 24 h as a filled bar). SPR is on the left y-axis. Filled circles and connecting lines represent ICA-105665 plasma concentration (right y-axis). For ease of viewing, the x-axis is broken into two segments. The left represents predose to 12 h and the right represents 24–28 h postdose.

ICA-105665 at single doses of 100, 400, and 500 mg reduced the SPR values in 1 of 4, 2 of 4, and 4 of 6 subjects, respectively. An example of change in SPR on day 2 following administration of ICA-105665 compared to day 1 (placebo) is shown in Fig. 2. Eleven of 19 sessions in the six Cohorts were performed by site 1, with three responses among these sessions. Site 3 performed six sessions (three responses) and site 4 performed two sessions (one response).

The proportion of responders apparently increased with dose, although the number of patients in each cohort was small. Of note, one patient participated in three dose cohorts and exhibited a response at 100 and 400 mg. This patient had decreases in SPR at three time points after a 200 mg dose, but also had a relative increase 1 h after the dose, resulting in a “nonresponse” by the protocol criteria.

The duration of response appeared to increase with dose (Table 1). One patient had complete suppression of photosensitivity following a 400 mg dose of ICA-105665. Onset of this response began 2 h postdose and was maintained for 4.5 h. The maximum suppression of photosensitivity was achieved at 2 h postdose. This same patient had partial suppression of photosensitivity at the 100 mg dose level, with onset of response beginning 1 h postdose, maximum response at 1 h postdose, and response maintained for 6.9 h.

**Table 1:** Temporal measures in standard photosensitivity response (SPR) for each patient with a positive response

Dose cohort (mg)	Patient number	Start time of response (h)	Duration of response (h)
100	104	1.0	6.9
400	109^a^	2.0	4.5
	303	1.2	22.9
500	112	1.0	23.0
	304	1.3	6.9
	306	4.1	20.1
	401	1.1	4.1

Positive response was either complete suppression or partial response.

a Patient met both complete and partial suppression criteria. The temporal variables were calculated based on the first and last time point of positive response, and longest duration.

Partial suppression of photosensitivity was also experienced by five other individual patients (one subject, 400 mg; four subjects, 500 mg). For these subjects, onset of response began between 1 and 4.1 h postdose. The duration of response generally increased with dose and ranged from 4.1 to 23.0 h. One patient in the 400 mg cohort and two patients in the 500 mg cohort maintained partial suppression of photosensitivity for at least 20 h.

Responders were found among the subjects with concomitant AEDs of LEV/CLZ (one subject with complete response); TPM (1), LTG (1), or none (3).

Among the nonresponders, concomitant AEDs were: LEV/LTG (2), LTG (1), TPM (1), PB/PHT (1), or none (1).

### Pharmacokinetics

**Figure 3:**
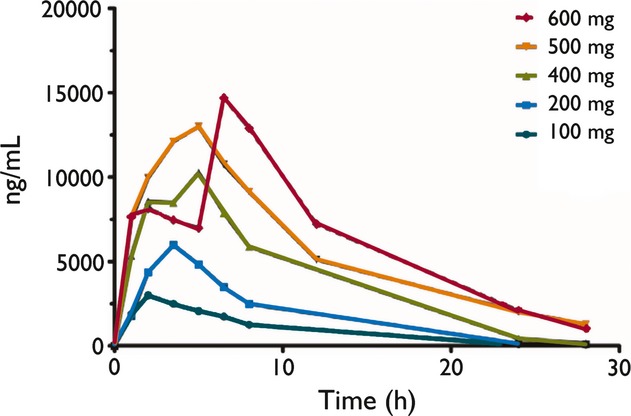
ICA-105665 plasma concentration by time for each dose group in study ICA-105665-04. Note that only one subject in the 600-mg dose group received ICA-105665.

Pharmacokinetic parameters are summarized in Table 2 and mean plasma concentration by time for each dose cohort is presented graphically in Figure 3. Maximum plasma concentration (C_max_, mean) increased with increasing dose as did area under the concentration-time curve to 24 h (AUC_24_). Mean time to maximum plasma concentration (T_max_) ranged from 2 to 6.5.

**Table 2:** Summary of mean (SD) noncompartmental pharmacokinetic parameters for ICA-105665 by treatment group^a^

Dose, mg (N)	C_max_, ng/ml	T_max_, h	Half-life, h
100 (4)	3,318 (873)	2.20 (0.73)	4.26 (0.91)
200 (4)	6,228 (2,073)	3.38 (1.23)	4.03 (0.81)
400 (4)	10,738 (3,856)	2.68 (1.44)	4.67 (1.90)
500 (6)	13,902 (3,932)	4.70 (1.91)	8.28 (4.04)
600 (1)	14,700	6.50	5.81

a Four subjects participated in more than one dose group. Subject 101, a female in the 100-mg dose group, was reenrolled as subject 105 in the 200-mg dose group. Subject 102, a male in the 100-mg dose group was reenrolled as subject 107 in the 200-mg dose group, and 108 in the 400-mg dose group. Subject 104, a male in the 100-mg dose group, was reenrolled as subject 106 in the 200-mg dose group and subject 109 in the 400 mg dose group. Subject 301 in the 200-mg dose group was reenrolled as subject 302 in the 400-mg dose group.

**Figure 4:**
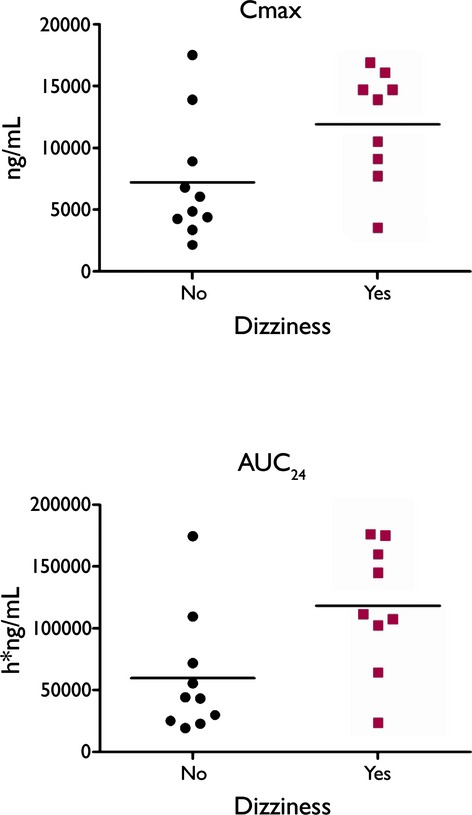
Comparison of C_max_ and AUC_24_ by presence (Yes) or absence (No) of dizziness as a reported AE (adverse event). Filled circles represent individual subjects that did not report dizziness. Filled squares represent subjects that did report dizziness. Lines represent mean values for each group.

Both C_max_ and AUC_24_ appeared to be higher in Responders (Fig. 4) compared to Nonresponders; however, the differences did not reach statistical significance (post hoc *t*-test). Time to maximal SPR suppression correlated with T_max_ in Responders (R^2^ = 0.8). However, maximal SPR suppression occasionally occurred long after T_max_.

### Safety and tolerability

The most common treatment emergent adverse events (TEAEs) following ICA-105665 administration were those related to the nervous system and included dizziness, somnolence, ataxia, and tremor (Table 3). The frequencies of TEAEs were higher among patients receiving the 500 mg dose compared to those at lower doses. Only one patient was studied at the 600-mg dose. This patient developed a brief generalized seizure within 1 h of dosing, leading to the discontinuation of additional patients at this dose, per the predefined protocol stopping rules. This patient, a 29-year-old white female with juvenile myoclonic epilepsy (JME) with TPM, was dosed with ICA-105665 after her day 2 baseline photic stimulation approximately 1 h after dosing; she was awakened and moved from a recumbent position to a chair for study-related phlebotomy. Prior to any photic stimulation, the patient reported dizziness, developed an episode of approximately 30 s of jerks of all extremities. At Screen the TPM concentration was 21.4 μg/ml, but on day −1 was 11.5 μg/ml. Although the patient had a history of generalized tonic–clonic seizure (GTCS) and myoclonic seizures (M), her last GTCS was 8 years prior to our study. In the investigator's opinion, this serious (medically significant) event was considered to be of moderate severity and possibly related to the study medication.

**Table 3:** Incidence of treatment emergent adverse events that occurred in two or more patients while receiving active treatment (safety population)

System organ class preferred term	Placebo (N = 13) n (%)	100 mg (N = 4) n (%)	200 mg (N = 4) n (%)	400 mg (N = 4) n (%)	500 mg (N = 6) n (%)	600 mg (N = 1) n (%)	Total (N = 13) n (%)
Any TEAE	4 (31)	1 (25)	0	2 (50)	6 (100)	1 (100)	10 (77)
Nervous system disorders	2 (15)	1 (25)	0	2 (50)	5 (83)	1 (100)	9 (69)
Dizziness	1 (8)	1 (25)	0	2 (50)	5 (83)	1 (100)	9 (69)
Somnolence	0	1 (25)	0	0	4 (67)	0	5 (38)
Ataxia	0	0	0	0	2 (33)	0	2 (15)
Tremor	0	0	0	0	2 (33)	0	2 (15)
General disorders and administration site conditions	0	0	0	0	2 (33)	1 (100)	3 (23)
Fatigue	0	0	0	0	2 (33)	1 (100)	3 (23)
Musculoskeletal and connective tissue disorders	0	0	0	0	2 (33)	0	2 (15)
Muscle twitching	0	0	0	0	2 (33)	0	2 (15)

AE, adverse event; MedDRA, medical dictionary for regulatory activities; TEAE, treatment-emergent adverse event.

TEAEs were attributed to ICA-105665 for any AEs starting or worsening on or after the date and time of ICA-105665 dose on day 2. TEAEs were attributed to placebo for any AEs occurring before ICA-105665 dose on day 2, but after placebo dose on day 1. Each patient was counted only once in the Total column for patients returning for a higher dose. At each level of patient summarization, a patient was counted once if the patient reported multiple events within the same Preferred Term of System Organ Class. System Organ Classes and Preferred Terms were coded using the Medical Dictionary for Regulatory Activities (MedDRA) version 12.1 (Italian Publishers Association, Milan, Italy).

All TEAEs at doses of 400 mg and lower were considered mild. At the 500-mg dose, dizziness, somnolence, and fatigue were rated moderate.

Dizziness was the adverse event that seemed to be most related to ICA-105665 administration (Table 3). Exposure to ICA-105665 was compared between subjects reporting dizziness and not reporting dizziness in Fig. 4. Both C_max_ (p = 0.0418) and AUC_24_ (p = 0.0209) were higher in subjects with dizziness compared to subjects not reporting dizziness.

## Discussion

The photosensitive epilepsy model has been used to evaluate investigational AEDs. This assay tries to provide a means of assessing CNS penetration, dose-dependent efficacy, and tolerability of an investigational drug following single-dose administration (Binnie et al., 1). Many drugs have been investigated in the last 20 years, including LEV, CBZ liquid formulation, and VGB, although certainly not all AEDs on the market have been tested. So far only one experimental drug, an NMDA full antagonist, Org6370, has proven not to be suppressive in a dose-dependent way, but proconvulsive (Kasteleijn-Nolst Trenité et al., 4). The findings with Icagen do unfortunately thus not predict efficacy in a specific subtype of epilepsy. It warrants further studies in epilepsy.

ICA-105665 was the first activator of neuronal Kv7 potassium channels tested for activity in the photosensitive epilepsy model in humans. Retigabine (also named ezogabine), another activator of Kv7 potassium channels (Rogawski & Bazil, 13), is effective for the treatment of partial seizures in humans (Rheims & Ryvlin, 8) and has been approved for use in Europe and the United States, but to the best of our knowledge has never been assessed in patients with photosensitive epilepsy or in patients with IGE. ICA-105665 decreased the SPR at single doses from 100 to 500 mg in individual subjects, with a higher proportion of subjects responding as the dose was increased. Duration of effect appeared to increase with increasing dose as well. No correlation with concomitant AED therapy was apparent from this small sample. Three subjects exhibited variability in their SPR that could have influenced the outcome. None of these subjects was classified as a responder in the analysis and in two subjects this variability was associated with spontaneous generalized epileptiform discharges, often resembling a PPR. None met the criteria for a proconvulsant effect, although in some eye conditions the response on day 2 appeared to be worse than day 1. In retrospect, and in future studies, patients with spontaneous discharges should be excluded.

As occurred in studies in healthy volunteers with ICA-105665, dizziness was the first emerging adverse event and the adverse event most related to ICA-105665 administration. Both efficacy and emergence of dizziness appeared to correlate with increasing plasma concentrations of ICA-105665.

Based on the efficacy and tolerability in the photosensitive epilepsy model, ICA-105665 should be assessed for the treatment of seizures in a more traditional, longer-term outpatient clinical trial to determine the molecule's utility for the treatment of epilepsy.
